# Endoscopic preperitoneal parastomal hernia repair (ePauli repair) : an observational study

**DOI:** 10.1007/s00464-020-08192-1

**Published:** 2021-01-04

**Authors:** Jan Roland Lambrecht

**Affiliations:** Department of Gastroenterological Surgery, Sykehuset Innlandet Hospital Trust, Furnesvegen 26, N-2380 Brumunddal, Norway

**Keywords:** Parastomal, Hernia, Repair, Ostomy, Laparoscopy, Robotic

## Abstract

**Background:**

Aspiring endoscopic surgery with extraperitoneal mesh application to avoid adhesion and pain from mesh fixation, we adopted the principles of the open Pauli repair of parastomal hernia (PSH). We have termed the procedure ePauli repair. The aim of this account is to inform about feasibility and adverse reactions.

**Methods:**

Patients with PSH selected for ePauli repair with transversus abdominis release (TAR) were enrolled in a prospective observational study. Patients were operated with laparoscopic or robotic assistance and endoscopic Rives-Stoppa repair in cases with concomitant midline hernia. Coated meshes or a buffer mesh was used in the retromuscular pocket for this modification of the Sugarbaker principle.

**Results:**

Fifteen patients were included: six patients were operated laparoscopically and nine patients with robotic assistance. The median age of the stomas was 33 months (7–313). Five PSHs were recurrent after previous repairs. Median operating time without midline hernia repair was 156 min (107–233) and with midline hernia repair 241 min (176–286). One serosa lesion arose during operation, prompting intraoperative revision of the ostomy without postoperative morbidity. Two patients had postoperative obstruction and were readmitted to operation: one with multiple adhesions and one had kinking of the stoma bowel caused by insufficient incision of the transversalis fascia. No infections or seromas have been observed. One patient had discoloring of the flank with spontaneous remission, and one patient had recurrence. Median postoperative admission time was 3 days (1–19). Median follow-up is 10 months (0–27).

**Conclusions:**

ePauli repair is technically challenging but feasible. With our limited experience, we are encouraged with the pain, complication, and functional summary after ePauli repair and hopeful for the recurrence profile. ePauli/TAR is not for every patient or every surgeon and whether it should be restrained to recurrent PSH or be offered as first-line treatment for PSH is disputable.

**Electronic supplementary material:**

The online version of this article (10.1007/s00464-020-08192-1) contains supplementary material, which is available to authorized users.

The near-invincible parastomal herniation (PSH) problem has been met with stoicism but is addressed more often with repair in recent years [1]. Historically, low efficiency rendered repairs to back-against-the-wall emergency situations. Recent mesh technology and technique have optimized recurrence rates and adverse outcomes – and a more aggressive attitude towards repair is exerted both from a patient and a surgeon perspective. A local repair with synthetic non-absorbable mesh has better outcomes than relocation. A laparoscopic intraperitoneal repair (IPOM) has lower infection rate compared to open repair. However, the one-year recurrence rate still seems to be considerable [2–4]. Key-hole techniques have worse outcome than the modified Sugarbaker technique: the most popular endoscopic procedure today with a recurrence rate around 15%.

E. Pauli has described a modification of the Sugarbaker technique [5], the “Pauli repair,” employing a transversus abdominis release (TAR) as described by E. Novitsky [6] with placement of mesh in the preperitoneal and/or pretransversalis fascia planes. Previous reports of TAR for incisional hernia with concurrent ostomy/PSH involved reinforced relocation or a key-hole repair with reconstruction of the stoma [7]. A series of open Pauli procedures have been published, with concerns about mesh complications and recurrence rate of 11% after a median of 13 months follow-up [8].

Intraperitoneal mesh increases risk of adhesion and fistula formation. In a quest for endoscopic repair with inherent less infection risk, but extraperitoneal mesh application to avoid adhesion and pain from mesh fixation, we adopted the principles of the Pauli repair to endoscopic surgery in a prospective series. We have termed the procedure ePauli repair. The aim of this account is to inform about feasibility and adverse reactions – and on longer-term basis also monitor recurrence rate.

## Materials and methods

### Inclusion

Patients with PSH selected for ePauli repair were enrolled in a prospective observational study. The study is registered as a local quality control study at Sykehuset Innlandet Hospital Trust with oral and written patient information and subscribed consent to participation and publication. The study and patient information are approved by the Institutional Review Board and the Data Protection Officer at Sykehuset Innlandet Hospital Trust with protocol ID 137157 and registered in ClinicalTrials.gov with ID NCT04440514.

### Surgery

Laparoscopy with three ports in the opposite flank, contralateral to the stoma/PSH. Robotic assistance was employed when available; however, in the beginning, patients with concomitant midline hernia were selected for a laparoscopic procedure. After adhesiolysis in the abdominal cavity and freeing of the intestinal stoma in the hernia cavity, extraperitoneal access (TAPP) was achieved by medial incision of the rectus sheath and a Rives dissection towards the semilunar line was performed, herewith incising the parastomal hernia sack along its circumference. Next step was TAR, with incision of the rectus sheath medial to the neurovascular bundles and medial release of the lamellae of the transversus abdominis muscle. An up-to-down approach was preferred as it is perceived easier to stay in the pretransversalis fascia plane and avoid making holes in the peritoneum during lateral development of the plane. The dissection was continued at least 10 cm lateral to the ostomy, and longitudinally to accommodate a 20 cm mesh. Previously placed meshes were left in situ. The posterior ostomy was moved by incision of the transversalis fascia, the bowel lateralized, and the fascia sutured medially. In the first patients, the mesh was held in place with sutures around the bowel – an inheritance from intraperitoneal Sugarbaker technique – alas, in the later procedures, we instead fixated the bowel to the flank with absorbable V-Loc, and we did not fixate the mesh. The anterior ostomy was adapted to accommodate the stoma bowel with non-absorbable V-Loc placing the bowel against the lateral edge of the opening. A mesh, typically 18 × 18 cm in size, was placed in the developed pocket – in front of the posterior fascia but behind the intestine and the anterior abdominal wall. Mesh choice was either Dynamesh with adhesion barrier anteriorly, or a uncoated synthetic midweight non-absorbable mesh with Bio-A synthetic absorbable mesh placed as barrier between the mesh and the bowel, laterally overlapping the non-absorbable mesh with 1 cm in order to avoid mesh ingrowth in the intestine. Ultimately the posterior rectus sheath was re-adapted to the linea alba with absorbable V-Loc. In patients with concomitant midline hernia planned for concurrent repair, the procedure was extended with an enhanced-view Rives-Stoppa (eRS) with port insertion in the ipsilateral flank and contralateral retromuscular dissection to the semilunar line. The linea alba was reconstructed with non-absorbable V-Loc and the posterior fascia/peritoneum closed with absorbable V-Loc. A mesh reaching from the contralateral semilunar line to the ipsilateral flank with stoma was placed in the retromuscular plane without fixation.

Edited video of a robotic ePauli is enclosed with this dynamic manuscript.

### Statistics

Descriptive statistics only.

## Results

Fifteen patients with ePauli repair for their PSH were included: six patients were operated with laparoscopy and nine patients with robotic assistance. Eleven PSHs were at end-colostomies, and three were at end-ileostomies and one at an urostomy. Ten of the colostomies and one of the ileostomies were after abdomino-perineal resection for rectal cancer. One colostomy was for anal incontinence, one ileostomy was after colectomy for ulcerative colitis, one ileostomy for constipation, and one urostomy was established for urinary incontinence. All ostomies had been formed penetrating the rectus muscle. Ten patients were male, and median age was 69 years (44–76). Mean body mass index was 27.6 kg/m^2^(SD 3.6), and the median age of the stomas was 33 months (7–313). Twelve patients had American Society of Anesthesiologist physical status score (ASA) of II and three patients ASA score III. Three patients were smokers, one had chronic obstructive pulmonary disease, and two had diabetes mellitus. None of the patients were on steroid therapy.

Five PSHs were recurrent after previous repairs: two suture repairs, one open retromuscular key-hole mesh repair, one open IPOM key-hole mesh repair, and one laparoscopic-modified Sugarbaker IPOM repair. The patient with open IPOM key-hole recurrence had received a retromuscular prophylactic polypropylene mesh at index operation as had the patient with laparoscopic-modified Sugarbaker IPOM repair and two of the patients with primary PSH. In two patients, a Dynamesh with barrier anteriorly towards the stoma bowel was used and in eleven cases a Optilene mesh, in one case a Versatex, and in one case a Parietex mesh, all non-coated meshes with inserts of a 8 × 8 cm Bio-A mesh to cushion the bowel. Four of the laparoscopic and three of the robotic cases had concomitant midline hernia repair that were repaired concurrently with PSH repair: six midline hernias were repaired with eRS whereas one of the robotic repairs was mended with IPOM+ (+ signifies linea alba reconstruction). One robotic case received additional stoma revision with skin plasty to reduce a greatly widened stoma opening with difficult stoma bag placement. Botox for lateral abdominal wall relaxation was not used in this series. Median operating time without accessory surgery beyond ePauli repair was 158 min (107–233) and with midline hernia repair 246 min (176–286). Robotic procedures were of insignificantly longer duration compared to laparoscopic procedures: median 168 (131–233) vs. 126 (107–145) minutes and 265 (236–278) vs. 233 (176–286) minutes, without and with concomitant midline hernia repair, respectively (*p* = 0.14 and *p* = 0.63, Mann–Whitney U tests).

In the recurrence after modified laparoscopic Sugarbaker IPOM repair, where a 15 × 15 cm parietex composite parastomal mesh had been used, we observed mesh rupture, and for this reason, this particular mesh has been revoked. In one of the primary PSHs with prophylactic mesh, we also observed lateral breakage of the mesh. In the remnant primary or recurrent PSH with key-hole type mesh prophylaxis or repair, the apertures were enlarged.

Perioperative complications arose in two patients: one serosal lesion in a laparoscopic procedure was sutured without further problems and one skin-near colotomy happened during adhesiolysis in a robotic procedure, which prompted a per operative revision of the ileostoma. This was the patient with IPOM+ repair of a concomitant midline hernia, which was not preoperatively planned for repair, but discovered significant and therefore addressed. This patient had much more postoperative pain than other patients with ePauli repair, and because of this a diagnostic laparoscopy was executed two days later without pathologic findings.

Postoperatively there were two patients with ileus – one had a coloscopy with normal findings, and the second resolved spontaneously after readmission and rehydration. Two patients developed obstruction, one of the small intestine and thus had a re-laparoscopy with adhesiolysis which was released. However, omentum was found herniating through the internal lateralized ostomy and was reduced, and the mesh incised. This patient is the only patient in the cohort so far with a postoperative bulge at the stoma site and with clinical recurrence, but it is small, unchanged in size since operation and causes no problems currently after 20 months. No other hernia recurrence in the midline or at the stoma has been observed. The other patient with obstruction, which was the first in the series, was at the edge of the mesh, but not mesh related – it was rather an insufficient lateral incision of the posterior fascia which kinked the bowel for being drawn medially after midline adaptation of the posterior fascia, thus having a re-laparoscopy with incision of the transversalis fascia laterally. The same patient had mesh fixation with transfascial sutures on each side of the colon at the lateral edge of the mesh and pain at the suture sites. After transcutaneous removal of sutures, the pain receded, and no patients have chronic pain after the procedures. Another postoperative complication was one patient with dark coloring of the flank, i.e., a hematoma which resolved spontaneously. None of the patients had drainage and no other bleeding, seroma, or infections have been observed. Only the patient with suture related pain and transcutaneous suture removal has been to the outpatient clinic for treatment of complications. One patient has not regained the ability to irrigate the ostomy and has intermittent pain and irregular stoma function. The CT shows a slightly curved retromuscular course of the intestine. Median postoperative admission was 3 days (1–19), and one patient was readmitted. Median follow-up is 10 months (0–27).

## Discussion

The endoscopic TAR applied for PSH has only recently been proposed, and in the European Hernia Society guidelines from 2018, the method was not assessed [9]. The concept was barely conceived, and there is little evidence of open application and no evidence of endoscopic application. Beyond congress reports and showcases, the endoscopic Pauli (ePauli) repair is only reported in peer-reviewed literature in a publication from Belyansky et al. as enhanced-view totally extraperitoneal repair (eTEP) [10], which is technically possible and may have advantages not dealing with intraabdominal adhesions but likely more perilous for adhesiolysis of the intestine in the parastomal hernia sack. Recently, a case report of a transabdominal procedure has been reported [11].

TAR for large and lateral hernias has become an invaluable instrument in our toolbox and increasingly performed by laparoscopy and robotically. Endoscopic anterior component separation is now rarely done, generally not only because of adoption of botulinum toxin for relaxation of the lateral abdominal muscles but also because of the TAR approach readily available in the already developed dissection plane when component separation is desirable. Application of TAR for the lateral PSH was a rational next step. In PSH repair, we believe it is important to release the stoma bowel completely from the hernia sac with the aim for the bowel to go straight through the subcutaneous and muscle layers of the abdominal wall so that irrigation is possible and no fecal obstruction or prominence from a convoluted bowel occurs. This adhesiolysis is challenging and led to two peroperative bowel lesions. One needed a stoma revision; however, there was no attached morbidity in these cases, and we do not perceive this as particularly associated to the Pauli eTAR modification of repair. This is also not aberrant from our experience with laparoscopic-modified Sugarbaker IPOM repair although damage that requires stoma revision is rare. The patient with suture-fixation pain was resolved, and no other patient had chronic pain and generally truly little pain is observed after eRS/ePauli repairs, much less than we perceive with the IPOM technique. Hematoma is a known complication also in our eTEP/eRS/eTAR experience – as in open Rives-Stoppa and TAR but we still only use drains selectively when performing endoscopic TEP and TAR. In our series of endoscopic transabdominal Pauli repairs, we cannot yet assess the true recurrence rate.

In one of the two obstruction cases, incision of the transversalis fascia as far as the “TAR plane” is dissected was not achieved and this resulted in kinking of the bowel for being drawn medially after midline adaptation of the fascia. This was fortunately easily resolved with complemental fascia incision. It is, therefore, imperative to fully incise the transversalis fascia as far lateral as the plane is dissected when lateralizing the inner ostomy. The other obstruction was a recurrent PSH after open abdomino-perineal rectum amputation with prophylactic stoma mesh and open key-hole repair. Adhesions were abundant and obstruction was linked to re-adhesion, however, we did observe omentum herniating into the inner aperture - so evidently ePauli is also not a fail-safe procedure in regard of incarceration or recurrence. A related possible caveat is rupture of the posterior fascia, which may lead to incarceration into the retromuscular space. This has not been observed in the study or in our eTEP/eTAR experience, but closure of the posterior fascia/peritoneum must be meticulous, and tension avoided. In ePauli repair and repairing a concomitant midline hernia, a two-sided access is necessary with TAPP approach as described in this report. Redocking when using robotic assistance is simplified with the Da Vinci Xi robotic system. With concomitant midline hernia repair, the eTEP approach with inherent preperitoneal crossover behind linea alba has the advantage of a contralateral one-sided access. However, we are fearful of exposure limitations and increased risk of bowel lesions during PSH sack adhesiolysis and have abstained from eTEP approach in PSH repair for this reason. To assuage the risk of mesh erosion into the stoma bowel as reported by Tataldi et al. [8], we have chosen to use a barrier or cushion mesh in Pauli repairs – in both open and endoscopic cases. The efficacy of this remedy is undetermined.

ePauli repair is a technically challenging endeavor, and profound knowledge of the anatomy and pathoanatomy of the abdominal wall is required. The procedure is feasible both in laparoscopic setting and with robotic assistance. To compare duration of procedure modalities in such a small sample is not sustainable and despite reaching a relatively standardized procedure, the variation in difficulty of each single case regarding adhesions and fibrosis creates substantial confounding. We perceive that exposure of the operative field, dissection – especially in the hernia sack - and suturing is more enabling and delicate with robotic assistance and provides improved agility and precision. Thus, robotic repair is perceived as safer and less strenuous, but we do not have evidence to suggest better outcome for the patients nor can substantiate cost effectiveness.

With our limited experience in fifteen patients, we are encouraged with the pain, complication, and functional summary of our patient population after ePauli repair, and we are hopeful that the recurrence profile also will be promising. ePauli/TAR is not for every patient or every surgeon [12]. The case load per surgeon for PSH repair in Norway is extremely low. Dedication and likely also case volume are important measures to obtain good results. Many PSH patients are waiting for surgery, and while standing in line, some are victims of emergency repair with much worse outcome. Not every PSH should be repaired, but patients with PSH-related problems where repair is contemplated should be referred to dedicated surgeons and prioritized. This would also facilitate studies to obtain real knowledge. Because of the complexity of ePauli repair is it disputable whether it should be restrained to recurrent PSH or be offered as first-line treatment. The ePauli repair is less applicable in patients with previous intraperitoneal or retromuscular mesh repair of midline hernia where a Sugarbaker approach might be a better choice – or in high-risk patients with massive adhesions or major disease where a local mesh repair is preferred. ePauli is viable in primary PSH and recurrences in patients with intraperitoneal, retromuscular, or on-lay mesh inserts for prophylaxis or repair if adequate skills are available at the treatment facility. Despite the theoretical advantages with ePauli, forthcoming evidence is required to demonstrate if our expectations of the method will be assured.

## Electronic supplementary material

Below is the link to the electronic supplementary material.

Supplementary file1 (MP4 617490 kb)
